# Anti-Gamma Aminobutyric Acid B (GABA B) Antibody Receptor-Associated Autoimmune Encephalitis in Pregnancy: A Rare Condition

**DOI:** 10.7759/cureus.73494

**Published:** 2024-11-11

**Authors:** Md Aziz Mazumdar, Sonali Bansal, Isha Shukla

**Affiliations:** 1 Medicine, Park Hospital, Gurgaon, IND; 2 Critical Care Medicine, Park Hospital, Gurgaon, IND; 3 Neurology, Park Hospital, Gurgaon, IND

**Keywords:** acute encephalitis, anti gaba-b antibody receptor, autoimmune limbic encephalitis, epilepsy in pregnancy, seizures in pregnancy

## Abstract

We report a primigravida 31-year-old female patient hospitalized at 32 gestational weeks with status epilepticus. In due course of illness, she developed refractory status epilepticus managed with induced coma with propofol and emergency early lower section caesarean surgery (LSCS). A battery of initial laboratory and radiological tests did not lead to a definite diagnosis. On further workup, she was diagnosed with anti-gamma aminobutyric acid B (GABA B) receptor autoimmune encephalitis that responded to immunotherapy and pulse steroids.

## Introduction

Anti-gamma aminobutyric acid B receptor (anti-GABA B) encephalitis, first described by Lancaster et al. in 2010, is a relatively rare condition, constituting approximately 5% of all known autoimmune encephalitis syndromes​ [1]. The condition primarily presents as limbic encephalitis, with hallmark symptoms including seizures, cognitive impairment, confusion, and personality changes. Less commonly, patients may experience cerebellar ataxia and opsoclonus-myoclonus syndrome​ [2]. Anti-GABA B receptor encephalitis is often associated with tumors, particularly small-cell lung cancer, with nearly half of all cases presenting alongside neoplasms​ [3]. Treatment strategies for this condition, including immunotherapies such as intravenous immunoglobulins, plasma exchange, corticosteroids, and tumor resection, remain debated​ [3]. This condition is linked to a high mortality rate, often due to tumor progression, and long-term complications such as seizures, memory deficits, and psychiatric disorders are common​ [4].

In pregnant women, the most common cause of seizures is eclampsia, followed by conditions like infections, posterior reversible encephalopathy syndrome, cortical vein thrombosis, and autoimmune encephalitis, particularly the anti-N-methyl-D-aspartate (NMDA) receptor subtype​ [5]. In this report, we describe a rare case of anti-GABA B receptor encephalitis in a pregnant woman who responded to immunotherapy and corticosteroids. To our knowledge, this is the first case of its kind reported during pregnancy in India.

## Case presentation

A 31-year-old primigravida, at 32 weeks of gestation, was admitted to the hospital with a history of generalized tonic-clonic seizures (GTCS) that had been recurring over the past 10 days. Her pregnancy had been uneventful until this point, with regular antenatal checkups. There was no apparent history of any cognition defect or any psychiatry disorder during the antenatal period, but there were some psychiatric symptoms for the past five days before admission in the form of intermittent abnormal behaviour and suspiciousness (delusions) associated with unaffected cognition. Upon admission, she was in a postictal state, having experienced a GTCS episode at home that lasted less than one minute, with no evidence of tongue biting or aspiration​. Her systolic blood pressure was 130 mmHg, and a trans-abdominal ultrasound confirmed a live fetus with normal anatomy and appropriate growth for gestational age.

**Figure 1:**
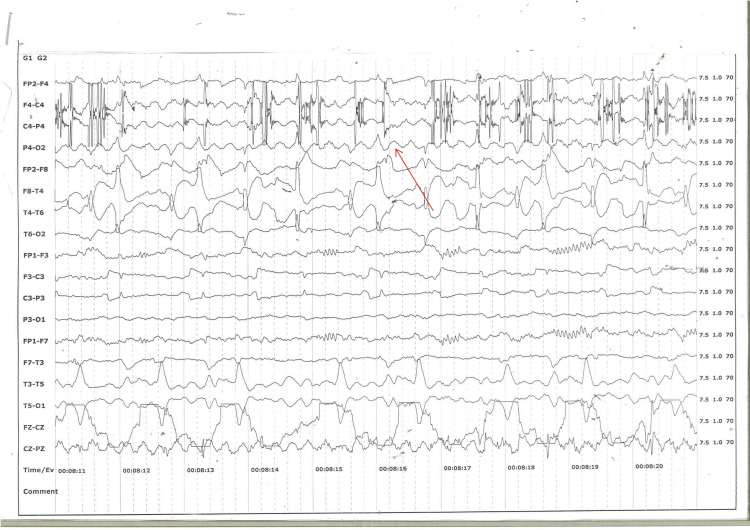
EEG showing generalised non convulsive status epilepticus Electroencephalogram (EEG), longitudinal montage, sensitivity 10 mcv/mm, background slowing seen, continuous spike and wave discharges seen, arising from right the fronto-centro parietal area, suggestive of non-convulsive status epilepticus.

**Figure 2:**
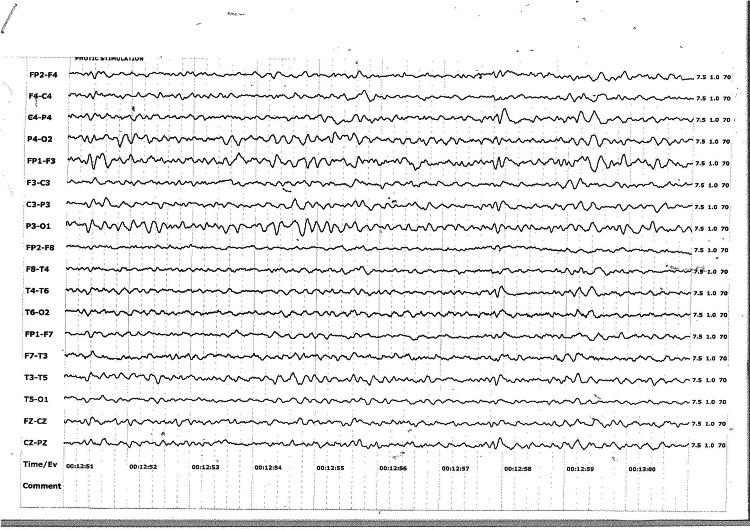
EEG showing generalised slowing after midazolam Electroencephalogram (EEG), longitudinal montage, sensitivity,7.5 mcv/mm. EEG showing generalized slowing, 5-6 Hz, 30-40 mcv theta activity. No epileptiform discharges seen.

Steroids (betamethasone 12 mg intramuscularly 24 hours apart) were administered to promote fetal lung maturity, and her urinary protein levels were found to be negative. Brain MRI showed no abnormalities, and cerebrospinal fluid (CSF) analysis revealed a pleocytosis and lymphocytic predominance with normal protein, glucose, and lactate levels. Blood, urine, and CSF cultures were negative, as was toxicology screening​. Initial investigations, including an electroencephalogram (EEG), revealed generalized seizures and non-convulsive status epilepticus (Figure 1). The seizures responded to intravenous lorazepam 4 mg intravenously and magnesium sulfate (Zuspan regime) (Figure 2).

On the second day of her hospital stay, the patient developed status epilepticus. Despite treatment with intravenous lorazepam 4 mg intravenously and levetiracetam 40 mg/kg intravenously, her condition did not improve, necessitating intubation and the initiation of propofol infusion (40 mcg/kg/min) with continuous EEG monitoring. An emergency lower segment cesarean section (LSCS) was performed, delivering a live male infant with low birth weight, who was transferred to the neonatal intensive care unit (NICU). Bilateral ovarian wedge resections were also performed, although histological analysis showed no abnormalities​.

Further diagnostic workup, including repeated lumbar punctures and CSF analysis, revealed the presence of anti-GABA B receptor antibodies in both the serum and CSF, confirming the diagnosis of autoimmune encephalitis​. The patient was treated with intravenous methylprednisolone (1000 mg/day) and intravenous immunoglobulins (0.4 g/kg/day) for five days. She was successfully weaned off propofol by the fourth day of hospital admission, and her seizures ceased. She was continued on oral levetiracetam 1000 mg per day. However, at two weeks of follow-up, while seizure-free, she exhibited poor long-term memory, inability to recognize family members, and intermittent psychiatric symptoms such as aggression and apathy towards her child​. At six months follow-up, the patient remained seizure-free, though she continued to experience cognitive deficits and psychiatric symptoms​.

## Discussion

This case represents the first reported instance of anti-GABA B receptor encephalitis in a pregnant woman from India​ [5,6]. It underscores the importance of considering autoimmune encephalitis in the differential diagnosis for pregnant patients with unexplained encephalopathy and seizures, as it can easily be misdiagnosed as eclampsia​ [4]. Typically, anti-GABA B receptor encephalitis affects middle-aged and elderly individuals and is frequently associated with tumors, particularly small-cell lung carcinoma​ [4]. The presentation of limbic encephalitis, marked by severe seizures, disorientation, and memory loss, is characteristic. EEG findings often reveal subclinical seizures or status epilepticus, and MRI may show medial temporal lobe involvement, though some patients exhibit normal imaging results​ [7].

In this case, the detection of anti-GABA B antibodies in both the serum and CSF allowed for the timely diagnosis and appropriate treatment with immunotherapy. Early intervention with corticosteroids and intravenous immunoglobulins likely contributed to the patient's recovery, though long-term cognitive and psychiatric issues persisted​ [8].

Autoimmune encephalitis caused by anti-NMDA receptor antibodies is the most common form of autoimmune encephalitis during pregnancy, and clinical manifestations are often attributed to eclampsia​ [9]. In cases like this one, testing for anti-GABA B receptor antibodies and other autoantibodies can aid in distinguishing autoimmune encephalitis from other more common causes of seizures during pregnancy, enabling early and effective treatment​ [10].

## Conclusions

Anti-GABA B receptor antibody-associated autoimmune encephalitis can be considered as a potential cause of autoimmune encephalitis in addition to anti-NMDA receptor antibodies autoimmune encephalitis during pregnancy. It is important to suspect this entity in a case of refractory seizures during pregnancy. Early treatment may bring favourable results. Such patients require good intensive care and interdisciplinary management. Such cases go unnoticed due to their very exclusive and rare findings associated with very overlapping clinical features with other neurological disorders, especially in relation to pregnancy, where the focus is mainly on eclampsia or other pregnancy-related complications.

The take-home message is the necessity for healthcare providers to suspect cases of autoimmune encephalitis should include rare subtypes like anti-GABA B receptor encephalitis, especially in pregnant patients with uncontrolled seizures, as it could be life-saving and prevent long-term cognitive and psychiatric disabilities.
